# Lateral-flow urine lipoarabinomannan for TB diagnosis in children

**DOI:** 10.5588/ijtldopen.25.0401

**Published:** 2026-03-13

**Authors:** R. Mahajan, L.F. Nyikayo, Y.B. Peter Ajack, B.T. Chol, M. Sangma, J. Ayor, M.J. Sagrado, A.E. Llosa, L. Moretó-Planas

**Affiliations:** 1 Médecins Sans Frontières, New Delhi, India;; 2 Médecins Sans Frontières, Malakal, Republic of South Sudan;; 3 Médecins Sans Frontières, Juba, Republic of South Sudan;; 4 Médecins Sans Frontières, Nairobi, Kenya;; 5 National Tuberculosis Program, Ministry of Health, Juba, Republic of South Sudan;; 6 Médecins Sans Frontières, Barcelona, Spain.

**Keywords:** tuberculosis, Republic of South Sudan, paediatric TB, LAM, MSF, malnutrition

## Abstract

**BACKGROUND:**

Diagnosing childhood TB is challenging due to nonspecific symptoms and difficulty obtaining sputum samples. This study evaluated the urine-based Alere Determine™ TB-LAM Ag test (AlereLAM) in a high-burden TB, HIV, and malnutrition setting.

**METHODS:**

Médecins Sans Frontières conducted a cross-sectional study in Malakal, South Sudan (October 2021–November 2023). Children (6 months–15 years) with presumptive TB received clinical and laboratory tests, including Xpert-Ultra and AlereLAM, regardless of HIV status. TB was classified as confirmed (Xpert-Ultra positive), unconfirmed (clinical), or unlikely.

**RESULTS:**

Of the 276 children (median age: 44 months), 53.3% (147/276) were female, 64.9% (179/276) were severely malnourished, and 9.4% (26/276) were children living with HIV. TB was confirmed in 10.5% (29/276), unconfirmed in 50.7% (140/276), and unlikely in 38.8% (169/276). Overall, AlereLAM positivity was 17.8% (49/276), with higher positivity in confirmed TB (27.6%; 8/29) than unconfirmed (20.0%; 28/140) and unlikely TB (12.1%; 13/107). Using confirmed plus unconfirmed TB as positive and unlikely TB as negative reference standard, sensitivity was 21.3% (95% confidence interval [CI]: 15.4–28.3), specificity 87.9% (95% CI: 80.1–93.4), positive predictive value (PPV) 73.5% (95% CI: 58.9–85.1), and negative predictive value 41.4% (95% CI: 34.9–48.1).

**CONCLUSION:**

AlereLAM’s high specificity and PPV support ruling in TB in resource-limited settings, but low sensitivity highlights the need for additional diagnostic tests.

TB remains a significant global health concern, particularly affecting vulnerable populations such as children and young adolescents. Despite ongoing efforts, diagnosing paediatric TB is difficult, leading to underreporting and delayed treatment. In 2023, the World Health Organization (WHO) estimated 1.3 million TB cases and 1,91,000 deaths among children aged 0–14 years.^1^ Alarmingly, over half of the TB cases in children and young adolescents went either undiagnosed or unreported. The estimated TB case detection rate was 41% in children aged 0–4 years and 63% in those aged 5–14 years and varied widely across countries.^2^ Diagnosing TB in children is particularly challenging due to the nonspecific nature of symptoms, which often mimic other common childhood illnesses. Paediatric TB cases are typically paucibacillary, making conventional diagnostics such as smear microscopy, molecular WHO-recommended rapid diagnostic tests (mWRDs), and culture less sensitive.^3–5^ Collecting suitable specimens is also difficult, particularly in young children who cannot produce sputum, and often requires invasive methods like gastric aspirates or induced sputum. This highlights the need for non-invasive diagnostic alternatives.^6^ TB and severe acute malnutrition (SAM) often co-occur in children, greatly increasing mortality risk.^7^ Similarly, TB-HIV co-infection remains a major contributor to paediatric mortality.^8^

The Alere Determine™ TB-LAM antigen test (AlereLAM; Abbott Laboratories, Palatine, IL, USA) is a low-cost (∼$3.5), urine-based point-of-care test that detects lipoarabinomannan (LAM), a marker of active TB. In use since 2015, it is simple for trained staff to perform, even in peripheral facilities, and delivers results within 30 min. AlereLAM was the first commercially available lateral-flow urine lipoarabinomannan (LF-LAM) assay, and is recommended by the WHO to assist in diagnosing active TB in adults, adolescents, and children living with HIV (CLHIV) who have TB symptoms, advanced HIV disease, are seriously ill, or have a CD4 count below 200 cells/mm^3^, regardless of symptoms.^9,10^ The latest WHO guideline recommends that in CLHIV with TB symptoms or screen positive, diagnosis should use both mWRDs and AlereLAM as the initial strategy, rather than mWRDs alone.^11^ The AlereLAM kit uses a grading card (Grade 1–4) for interpretation, with ≥Grade 1 considered positive. Some studies apply a higher threshold (≥Grade 2), reducing false positives but lowering sensitivity and increasing false negatives.^12,13^

Several high-TB- and HIV-burden countries (e.g., Chad, Zambia, Kenya, Malawi, and Uganda) have integrated LAM testing into national TB programmes.^14^ In Zambia, AlereLAM is also used in emergency settings, such as sepsis or SAM, even when a child’s HIV status is unknown. In a meta-analysis, the pooled sensitivity and specificity of LF-LAM were 49% (95% confidence interval [CI]: 48–50) and 90% (95% CI: 90–91), respectively, in adults irrespective of HIV status, and 37% (95% CI: 34–40) and 80% (95% CI: 79–82), respectively, in children.^15^ A separate meta-analysis reported pooled sensitivity and specificity of 46% (95% CI: 40–51) and 80% (95% CI: 69–91), respectively, among children aged <15 years. Among CLHIV, pooled sensitivity and specificity were 47% (95% CI: 33–60) and 77% (95% CI: 57–96), respectively, while in children without HIV, pooled sensitivity and specificity were 32% (95% CI: 8–57) and 79% (95% CI: 63–96), respectively.^10^ New LAM-based assays are being investigated to enhance TB diagnosis and treatment monitoring in both people living with HIV (PLHIV) and HIV-negative individuals.^16,17^ A few studies suggest a potential value of AlereLAM to diagnose TB in children with SAM.^12,13,18,19^ One study suggested that a ≥Grade 2 AlereLAM result could help identify TB in HIV-negative children with SAM, highlighting the need for further validation.^13^ In 2023, South Sudan reported a TB incidence of 227 per 100,000, with 17% of cases in children under 15.^20^ Since 2016, Médecins Sans Frontières (MSF) has supported TB diagnosis and treatment at Malakal, South Sudan. The South Sudan national TB programme recommends AlereLAM only for PLHIV with TB symptoms or low CD4 counts.

This study evaluates the performance of AlereLAM in children aged ≤15 years with presumptive TB in Malakal, irrespective of HIV status.

## METHODS

A cross-sectional study was conducted in South Sudan at Malakal teaching hospital and the Protection of Civilians (PoC) hospital in Malakal between October 2021 and November 2023. Malakal teaching hospital is a state-level facility (equivalent to a secondary hospital), while the PoC hospital functions at the county level (equivalent to a primary hospital).

### Study population, clinical, and laboratory procedures

Children aged 6 months to 15 years were classified as presumptive TB cases if they exhibited a persistent cough >2 weeks, unexplained fever >1 week, or signs of extra-pulmonary TB, including gibbous spinal deformity, lymphadenopathy, subacute meningitis, ascites, diarrhoea >2 weeks, painless joint swelling, or pleural effusion.

Additional presumptive TB cases were identified after 1 week of inpatient admission, defined by low weight gain despite nutritional therapy, persistent pneumonia or cough unresponsive to antibiotics, persistent fever (>38°C), and persistent fatigue. Screening included assessment of TB exposure, prior TB, and HIV status. Physical examinations included anthropometric measurements, and HIV testing was performed for children with unknown status. All children were tested with AlereLAM on urine and with Xpert MTB/RIF Ultra (Xpert-Ultra) on at least one pulmonary or extra-pulmonary specimen, along with stool and urine samples in accordance with the manufacturer’s instructions. Pulmonary specimens comprised nasopharyngeal aspirates, gastric lavage, and spontaneous sputum. Extra-pulmonary specimens included lymph node aspirates, pus aspirates, and ascitic, pleural, and cerebrospinal fluids. Further details on testing with Xpert-Ultra in a similar context are provided elsewhere.^21^ In line with consensus childhood TB definitions,^22^ children were classified by the treating physician as: 1) Confirmed TB – Xpert-Ultra positive on any pulmonary or extra-pulmonary sample; 2) Unconfirmed TB – clinical diagnosis without Xpert-Ultra confirmation, based on a decision algorithm (Supplementary Data S1); or 3) Unlikely TB – Xpert-Ultra negative with an alternative diagnosis responding to treatment, and TB therapy not initiated. Clinicians were blinded to AlereLAM results, except for CLHIV.

For programmatic reasons, TB treatment was initiated in all CLHIV who were AlereLAM positive and had TB symptoms or low CD4 counts. These cases were classified as unconfirmed TB unless confirmed by Xpert-Ultra.

### AlereLAM testing procedure

Urine for AlereLAM testing was collected using sterile urinary bags for infants and clean collection pots for older children. Unprocessed specimens were tested in the MSF laboratory in Malakal, following the manufacturer’s instructions, and results were read after 25–35 min. Specimens were transported within 10–15 min on average. The intensity of the result bar was graded using the manufacturer’s reference scale (Grade 1: lowest intensity and Grade 4: highest intensity). A bar intensity of grade ≥ 1 was considered positive. Laboratory staff were blinded to the clinical data.

### Data collection and analysis

As a secondary study in a routine programme setting, no formal sample size calculation was performed. All consecutively enrolled children were included. Data were collected using structured paper forms and entered into Research Electronic Data Capture (REDCap) software. All data were pseudonymised. Continuous variables were reported using means and standard deviations (SDs) or medians and interquartile ranges (IQRs) as appropriate, and categorical variables as frequencies and percentages. Age-appropriate anthropometric indicators were calculated using WHO growth standards. SAM was defined per WHO criteria: weight-for-height Z-score < −3 SD, MUAC < 115 mm, or bilateral oedema for children <5 years, and BMI-for-age Z-score < −3 SD for children aged 5–15 years.^23,24^

AlereLAM performance was evaluated using two reference standards. The primary reference (composite reference standard) grouped confirmed and unconfirmed TB as ‘TB-positive’, with the unlikely TB group as TB negative. The secondary reference (microbiological reference standard) considered only confirmed TB cases as positive. Sensitivity, specificity, positive predictive value (PPV), and negative predictive value (NPV) were calculated with Clopper–Pearson 95% CIs. Diagnostic yield was defined as the proportion of children testing positive with AlereLAM among all tested. All statistical differences, including subgroup differences in diagnostic accuracy, were tested in univariable analyses using χ², Fisher Exact, or Kruskal–Wallis rank sum tests, as appropriate. All estimates are presented with 95% CIs, and *P* values <0.05 were considered significant. Data were analysed using R software (version 4.3.2; The R Foundation, Vienna, Austria). The results are reported in accordance with the Standards for Reporting Diagnostic Accuracy Studies (STARD) guidelines (Supplementary Data S2).

### Ethical statement

Ethical approval was granted by the MSF Ethics Review Board (ID 18116) and South Sudan’s Ministry of Health (ID MOH/ERB 20/2019). Written informed consent was obtained from legal guardians, and assent was taken from children aged ≥10 years.

## RESULTS

Of 327 eligible children, 51 were not tested with AlereLAM due to stockouts, leaving 276 for analysis – 77.9% (215/276) from Malakal Teaching Hospital and 22.1% (61/276) from PoC Hospital. Participant flow is shown in Supplementary Data Figure S1. The median age of the children was 44 months (IQR, 18–96), 57.9% (160/276) were <5 years, 53.3% (147/276) were female, 64.9% (179/276) had SAM, and 9.4% (26/276) were CLHIV, of whom 11.5% (3/26) had CD4 <200 cells/mm^3^. TB categorisation included 10.5% (29/276) confirmed TB, 50.7% (140/276) unconfirmed TB, and 38.8% (107/276) unlikely TB.

Among TB-positive children (confirmed and unconfirmed, n = 169), 44.4% (75/169) had pulmonary TB, 24.3% (41/169) had extra-pulmonary TB, and 31.4% (53/169) had disseminated TB. TB-positive children were younger (median 36 vs. 60 months; *P* = 0.007), had lower mid-upper arm circumference (median 130 vs. 140 mm; *P* = 0.002), and a higher proportion were CLHIV (14.8% vs. 0.9%; *P* < 0.001) compared with children with unlikely TB. Baseline demographic and clinical characteristics by TB category are shown in Supplementary Data Table S1.

The overall AlereLAM yield was 17.8% (49/276), with 27.6% (8/29) in confirmed TB, 20.0% (28/140) in unconfirmed TB, and 12.1% (13/107) in unlikely TB. Using a threshold of ≥Grade 2, the overall yield decreased to 9.4% (26/276): 17.2% (5/29) in confirmed TB, 11.4% (16/140) in unconfirmed TB, and 4.7% (5/107) in unlikely TB. The overlap of TB-positive results across different specimen types using Xpert-Ultra and AlereLAM is shown in the Figure.

**Figure.**
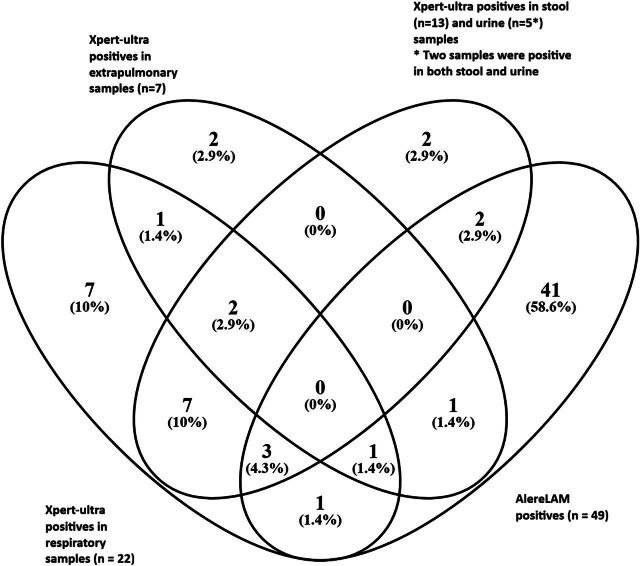
Overlap of TB-positive results detected by Xpert-Ultra and AlereLAM across different specimen types. AlereLAM = Alere Determine™ TB-LAM Ag test.

Using the composite reference standard, AlereLAM demonstrated a sensitivity of 21.3% (95% CI: 15.4–28.3), specificity of 87.9% (95% CI: 80.1–93.3), PPV of 73.5% (95% CI: 58.9–85.1), and NPV of 41.4% (95% CI: 34.9–48.1) (Table). Sensitivity was higher in children <5 years (29.9% vs. 6.5% in 5–15 years) and in CLHIV (60% vs. 14.6% in HIV-negative), while specificity did not differ significantly by age (81.1% in age< 5 vs. 94.4% in the 5–15-year) or HIV status (100% vs. 87.7%).Test performance did not differ by TB type or nutritional status (Table). Using a threshold of grade ≥ 2, specificity increased to 95.3% (95% CI: 89.4–98.5) and PPV to 80.8% (95% CI: 60.6–93.4), but sensitivity declined to 12.4% (95% CI: 7.9–18.4) and NPV remained unchanged at 40.8% (95% CI: 34.6–47.2). Using the microbiological reference standard, among 29 confirmed TB cases (Xpert-Ultra positive), sensitivity was 27.6% (95% CI: 12.7–47.2), with no significant differences across age groups, TB type, or nutritional status (Supplementary Data Table S2).

**Table.** AlereLAM performance using the composite reference standard.

	TP | FP | TN | FN	% sensitivity (95% CI)	% specificity (95% CI)	% PPV (95% CI)	% NPV (95% CI)
Overall (n = 276)	36 | 13 | 94 | 133	21.3 (15.4, 28.3)	87.9 (80.1, 93.4)	73.5 (58.9, 85.1)	41.4 (34.9, 48.1)
Age group					
<5 years (n = 160)	32 | 10 | 43 | 75	29.9 (21.4, 39.5)	81.1 (68, 90.6)	76.2 (60.5, 87.9)	36.4 (27.8, 45.8)
5–15 years (n = 116)	4 | 3 | 51 | 58	6.5 (1.8, 15.7)	94.4 (84.6, 98.8)	57.1 (18.4, 90.1)	46.8 (37.2, 56.6)
HIV status					
CLHIV (n = 26)	15 | 0 | 1 | 10	60.0 (38.7, 78.9)	100 (2.5, 100)	100 (78.2, 100)	9.1 (0.2, 41.3)
Without HIV (n = 250)	21 | 13 | 93 | 123	14.6 (9.3, 21.4)	87.7 (79.9, 93.3)	61.8 (43.6, 77.8)	43.1 (36.4, 49.9)
TB site^A^					
Pulmonary (n = 75)	14 | NE | NE | 61	18.7 (10.6, 29.3)			
Extra-pulmonary (n = 41)	8 | NE | NE | 33	19.5 (8.8, 34.9)			
Disseminated (n = 53)	14 | NE | NE | 39	26.4 (15.3, 40.3)			
Nutritional status					
SAM (n = 179)	26 | 11 | 54 | 88	22.8 (15.5, 31.6)	83.1 (71.7, 91.2)	70.3 (53.0, 84.1)	38.0 (30.0, 46.5)
Not SAM (n = 97)	10 | 2 | 40 | 45	18.2 (9.1, 30.9)	95.2 (83.8, 99.4)	83.3 (51.6, 97.9)	47.1 (36.1, 58.2)
With AlereLAM grade ≥ 2 as positive (n = 276)	21 | 5 | 102 | 148	12.4 (7.9, 18.4)	95.3 (89.4, 98.5)	80.8 (60.6, 93.4)	40.8 (34.6, 47.2)

AlereLAM = Alere Determine™ TB-LAM Ag test; TP = true positives; FP = false positives; TN = true negatives; FN = false negatives; n = number of samples; CI = confidence interval; CLHIV = children living with HIV; NE = not estimable; SAM = severely acute malnourished; PPV = positive predictive value; NPV = negative predictive value.

A Data available for only TB-positive children. Hence, only sensitivities could be calculated.

### Subgroup analysis of the cohort of SAM children without HIV

Among 160 children with SAM and without HIV, using the composite reference standard, AlereLAM sensitivity was 15.8% (15/95; 95% CI: 9.1–24.7) and specificity was 83.1% (54/65; 95% CI: 71.7–91.2). Raising the positivity threshold from grade ≥ 1 to grade ≥ 2 increased the PPV from 57.7% (15/26; 95% CI: 36.9–76.6) to 73.3% (11/15; 95% CI: 44.9–92.2), while sensitivity declined to 11.6% (95% CI: 5.9–19.8) and specificity rose to 93.8% (95% CI: 85.0–98.3). All children testing positive at Grades 3 or 4 were TB-positive (confirmed or unconfirmed).

## DISCUSSION

This study evaluated AlereLAM performance for TB diagnosis in one of the largest paediatric cohorts in a high-burden TB, HIV, and malnutrition setting. Using a composite reference standard, AlereLAM showed low sensitivity (21%) but high specificity (88%), while sensitivity increased slightly to 28% with a molecular reference standard. These findings are consistent with previous studies showing low sensitivity and high specificity in children, particularly in those without HIV.^10,15,18^

Our findings indicate that AlereLAM does not meet the WHO target product profile for sensitivity.^25^ Nevertheless, given the challenges of paediatric TB diagnosis and the lack of defined sensitivity thresholds for children, tools that support clinical decision-making remain valuable. The low sensitivity highlights limitations of AlereLAM as a standalone diagnostic tool, but its relatively high specificity (88%) and PPV (74%) indicate that most positive test results were correctly classified. Using a higher cut-off (≥grade 2) further improved specificity (95%) and PPV (81%), though sensitivity declined, consistent with Nicol et al.,^12^ who reported a similar trade-off.

Our study found that when using a composite reference standard, AlereLAM sensitivity was slightly higher in children under five and CLHIV, while specificity remained stable across age groups, TB presentation (pulmonary or extra-pulmonary), and nutritional status. However, the small number of CLHIV (n = 26) limits interpretation. All CLHIV with positive AlereLAM (n = 15) were started on TB treatment and classified as unconfirmed TB due to programmatic decisions, potentially overestimating sensitivity. None of the 26 CLHIV had TB confirmed by Xpert-Ultra, limiting sensitivity evaluation with the microbiological reference standard and reflecting the difficulty of microbiological confirmation in paucibacillary TB. Consistent with Schramm et al.,^13^ our study found that using an AlereLAM positivity cut-off of Grade ≥ 2 improved PPV in HIV-negative children with SAM from 57% to 73%. However, the small sample size and overlapping CIs limit the strength of this finding. Furthermore, reading AlereLAM cards is subjective, particularly distinguishing Grade 1 from Grade 2, which can lead to inter-reader variability.

Despite modest sensitivity, AlereLAM’s high specificity and PPV, rapid turnaround, and ease of use make it a valuable point-of-care test, particularly in resource-limited settings with limited access to diagnostics such as GeneXpert or X-ray. Paediatric TB diagnosis is challenged by the paucibacillary nature of the disease and difficulty obtaining adequate samples.^4,5^ In this context, urine-based tests like AlereLAM provide a non-invasive, practical alternative. In our clinics, AlereLAM proved feasible in a low-resource setting like Malakal, with minimal training requirements and suitability for routine use, although usability data were not formally collected.

Strengths of this study include a large sample size and inclusion of children from a routine programme setting, enhancing generalisability. Limitations include the small number of confirmed TB cases by Xpert-Ultra (n = 29), the cross-sectional design which precludes assessment of long-term outcomes, and lack of evaluation by disease severity. Use of imperfect reference standards, a common challenge in diagnostic evaluation studies in paediatric TB, is another limitation; with 50.7% (140/276) classified as unconfirmed TB, the composite reference standard may risk overdiagnosis, potentially affecting accuracy estimates.

Evidence indicates that AlereLAM has a measurable impact on mortality among PLHIV, particularly those with advanced immunosuppression, while paediatric data remain limited.^26,27^ In Kenya, LAM-positive CLHIV had nearly a five-fold higher risk of mortality than LAM-negative children.^28^ Therefore, we recommend early initiation of TB treatment in children testing positive on AlereLAM, particularly with a Grade ≥ 2 result, regardless of HIV or nutritional status, especially in settings lacking mWRDs or chest X-ray. Further programmatic and operational research is needed to validate these findings and explore ways to enhance the sensitivity of AlereLAM, including potential modifications to the assay or its use in combination with other tests.

## CONCLUSION

AlereLAM can serve as a useful adjunctive diagnostic tool for paediatric TB, particularly in resource-limited settings. Its high specificity and PPV support its role in ruling in TB, though low sensitivity necessitates additional diagnostic methods. Further research and development of next-generation LF-LAM tests are needed to improve performance, especially in children and HIV-negative individuals.

## Supplementary Material





## References

[1] World Health Organization. Global tuberculosis report 2024. Geveva: WHO, 2024.

[2] Yerramsetti S, Global estimates of paediatric tuberculosis incidence in 2013–19: a mathematical modelling analysis. Lancet Glob Health. 2022;10(2):e207-e215. 34895517 10.1016/S2214-109X(21)00462-9 PMC8800006

[3] Kunkel A, Smear positivity in paediatric and adult tuberculosis: systematic review and meta-analysis. BMC Infect Dis. 2016;16(1):282. 27296716 10.1186/s12879-016-1617-9 PMC4906576

[4] Reuter A, Hughes J, Furin J. Challenges and controversies in childhood tuberculosis. Lancet. 2019;394(10202):967-978. 31526740 10.1016/S0140-6736(19)32045-8

[5] Carvalho I. Tuberculosis in children: challenges in diagnosis and the decision to treat. Portuguese J Pediatr. 2022;53(4):671-673.

[6] Ioos V, Cordel H, Bonnet M. Alternative sputum collection methods for diagnosis of childhood intrathoracic tuberculosis: a systematic literature review. Arch Dis Child. 2019;104(7):629-635. 30127061 10.1136/archdischild-2018-315453

[7] Vonasek BJ, Tuberculosis in children with severe acute malnutrition. Expert Rev Respir Med. 2022;16(3):273-284. 35175880 10.1080/17476348.2022.2043747 PMC9280657

[8] Vonasek BJ, Tuberculosis in children living with HIV: ongoing progress and challenges. J Pediatr Infect Dis Soc. 2022;11(Suppl 3):S72-S78. 10.1093/jpids/piac060 36314545

[9] World Health Organization. Lateral flow urine lipoarabinomannan assay (LF-LAM) for the diagnosis of active tuberculosis in people living with HIV - policy update 2019. Geneva: WHO, 2019.

[10] Seid G, Value of urine-based lipoarabinomannan (LAM) antigen tests for diagnosing tuberculosis in children: systematic review and meta-analysis. IJID Reg. 2022;4:97-104. 35880002 10.1016/j.ijregi.2022.06.004 PMC9307507

[11] World Health Organization. WHO consolidated guidelines on tuberculosis. module 3: diagnosis. Geneva: WHO, 2025. 40388555

[12] Nicol MP, Accuracy of a novel urine test, Fujifilm SILVAMP tuberculosis lipoarabinomannan, for the diagnosis of pulmonary tuberculosis in children. Clin Infect Dis. 2021;72(9):E280-E288. 32761178 10.1093/cid/ciaa1052 PMC8096212

[13] Schramm B, Potential value of urine lateral-flow lipoarabinomannan (LAM) test for diagnosing tuberculosis among severely acute malnourished children. PLoS One. 2021;16(5):e0250933. 33951082 10.1371/journal.pone.0250933 PMC8099085

[14] Pai M, Adoption and uptake of the lateral flow urine LAM test in countries with high tuberculosis and HIV/AIDS burden: current landscape and barriers. Gates Open Res. 2020;4:24. 32185366 10.12688/gatesopenres.13112.1 PMC7059561

[15] Yin X, Diagnostic value of lipoarabinomannan antigen for detecting Mycobacterium tuberculosis in adults and children with or without HIV infection. J Clin Lab Anal. 2022;36(2):e24238. 35034374 10.1002/jcla.24238 PMC8842169

[16] Zhang Y, Breakthrough of chemiluminescence-based LAM urine test beyond HIV-positive individuals: clinical diagnostic value of pulmonary tuberculosis in the general population. Med (United States). 2023;102(48):e36371. 10.1097/MD.0000000000036371 PMC10695621 38050275

[17] Sigal GB, A novel sensitive immunoassay targeting the 5-methylthio-D-xylofuranose–lipoarabinomannan epitope meets the WHO’s performance target for tuberculosis diagnosis. J Clin Microbiol. 2018;56(12):e01338-18. 30257899 10.1128/JCM.01338-18 PMC6258851

[18] Nkereuwem E, Comparing accuracy of lipoarabinomannan urine tests for diagnosis of pulmonary tuberculosis in children from four African countries: a cross-sectional study. Lancet Infect Dis. 2021;21(3):376-384. 33316214 10.1016/S1473-3099(20)30598-3

[19] Osorio DV, Lipoarabinomannan antigen assay (TB-LAM) for diagnosing pulmonary tuberculosis in children with severe acute malnutrition in Mozambique. J Trop Pediatr. 2021;67(3):fmaa072. 33038897 10.1093/tropej/fmaa072 PMC8319630

[20] World Health Organization. Tuberculosis profile: South Sudan. Juba, South Sudan: WHO, 2024. https://worldhealthorg.shinyapps.io/tb_profiles/?_inputs_&tab=%22tables%22&lan=%22EN%22&iso2=%22SS%22&entity_type=%22country%22 (Accessed 23 May 2025).

[21] Moretó-Planas L, Xpert-ultra assay in stool and urine samples to improve tuberculosis diagnosis in children: the Médecins Sans Frontières experience in Guinea-Bissau and South Sudan. Open Forum Infect Dis. 2024;11(5):ofae221. 38798893 10.1093/ofid/ofae221 PMC11119760

[22] Graham SM, Clinical case definitions for classification of intrathoracic tuberculosis in children: an update. Clin Infect Dis. 2015;61(Suppl 3):S179-S187. 10.1093/cid/civ581 PMC4583568 26409281

[23] de Onis M, Development of a WHO growth reference for school-aged children and adolescents. Bull World Health Organ. 2007;85(09):660-667. 18026621 10.2471/BLT.07.043497 PMC2636412

[24] de Onis M, Worldwide implementation of the WHO child growth standards. Public Health Nutr. 2012;15(9):1603-1610. 22717390 10.1017/S136898001200105X

[25] World Health Organization. Target product profiles for tuberculosis diagnosis and detection of drug resistance. Geneva: WHO, 2024. https://iris.who.int/bitstream/handle/10665/378358/9789240097698-eng.pdf?sequence=1 (Accessed 10 September 2025).

[26] Suwanpimolkul G, Utility of urine lipoarabinomannan (LAM) in diagnosing tuberculosis and predicting mortality with and without HIV: prospective TB cohort from the Thailand Big City TB Research Network. Int J Infect Dis. 2017;59:96-102. 28457751 10.1016/j.ijid.2017.04.017

[27] Gupta-Wright A, Detection of lipoarabinomannan (LAM) in urine is an independent predictor of mortality risk in patients receiving treatment for HIV-associated tuberculosis in sub-Saharan Africa: a systematic review and meta-analysis. BMC Med. 2016;14(1):1-11. 27007773 10.1186/s12916-016-0603-9 PMC4804532

[28] Lacourse SM, Urine tuberculosis lipoarabinomannan predicts mortality in hospitalized human immunodeficiency virus-infected children. Clin Infect Dis. 2018;66(11):1798-1801. 29324985 10.1093/cid/ciy011 PMC5961239

