# Supplementary material for: Lateral-flow urine lipoarabinomannan for TB diagnosis in children
Source: IJTLD Open. 2026 Mar 13;3(3):157–62. doi: 10.5588/ijtldopen.25.0401 (PMC12991601; doi:10.5588/ijtldopen.25.0401)
Supplement: Supplementary file 1 [file ijtldopen25-0401_supplementarydata1.pdf]

Lateral-flow urine lipoarabinomannan for TB diagnosis in children: a cross-sectional study in South Sudan

Supplementary material

Supplementary file 1

Médecins sans Frontières Standard Operating Procedure (SOP) for the diagnosis of TB in children

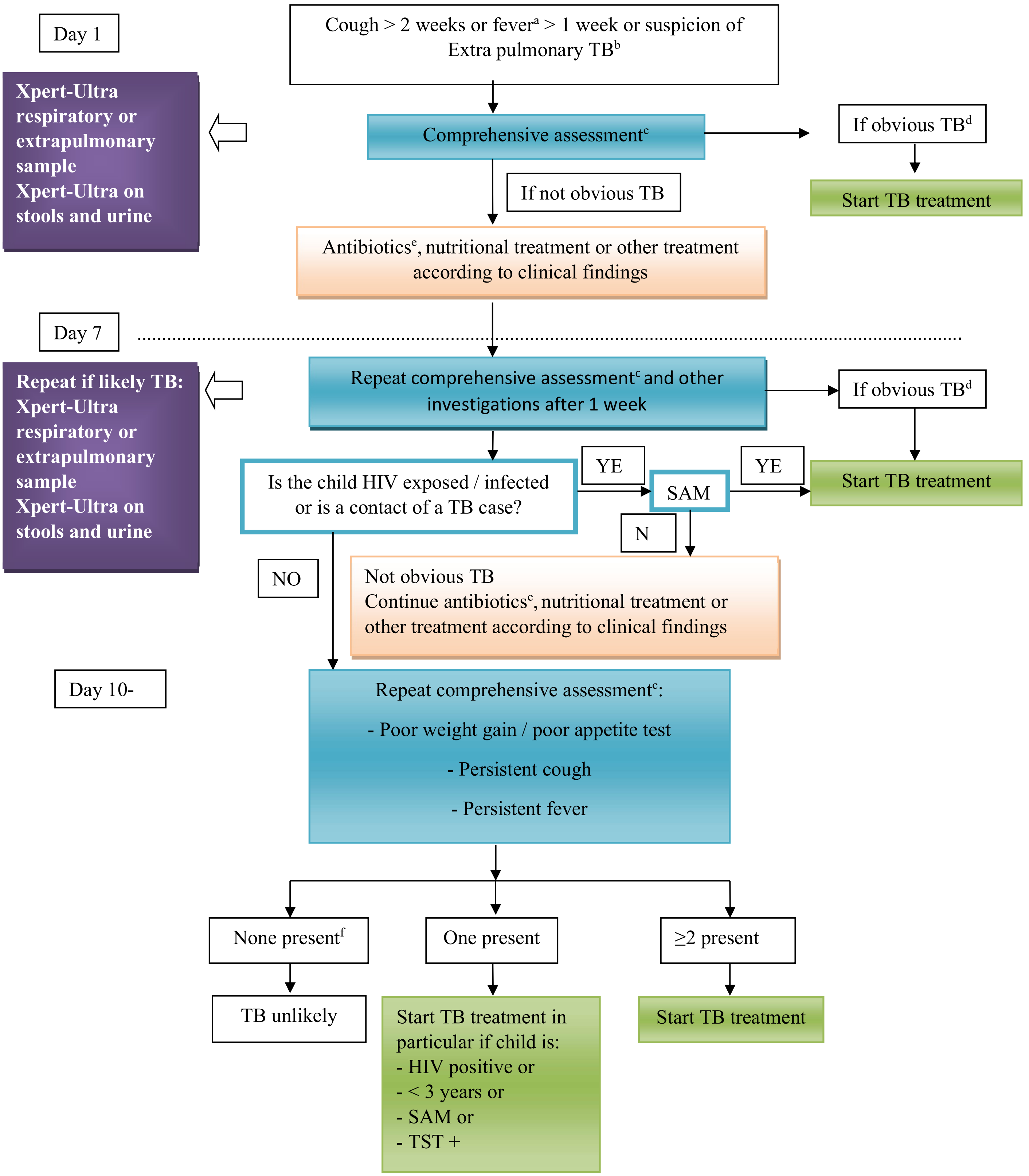

a) Axillar temperature > 38ºC
b) Enlarged lymph nodes, gibbous, > 2 weeks diarrhoea, meningitis, malnutrition not improving with proper nutritional treatment without other obvious causes (e.g. *Visceral leishmaniasis*)
c) Comprehensive assessment includes:
    a. Clinical assessment
    b. Growth assessment
    c. Bacteriological test (sputum collection, naso-pharyngeal aspirate, gastric lavage, lymph node aspiration):
        - Xpert MTB/RIF testing (sputum, nasopharyngeal aspirate, gastric lavage, lymph node aspirate, CSF);
        - Xpert MTB/RIF in stools and urine;
        - TB culture and DST (only at baseline):
        - Tb LAM for children living with HIV (CLHIV)

       For children:
        - < 5 years old: do nasopharyngeal aspirate or gastric lavage;
        - From 5 years old until child can expectorate: gastric lavage;
        - For children who can expectorate: sputum.
    d. POCUS (only at baseline)
    e. HIV testing if not yet performed
    f. When relevant and available: X-ray (CXR, spine)
d) Xpert MTB/RIF positive, CXR showing suggestive lesions (e.g. hilar lymphadenopathy, upper lobe infiltrates, and miliary picture), gibbous.
e) Broad spectrum antibiotics:
    - **If no signs of severity:**
        - First line: amoxicillin PO for 7 days (NO fluoroquinolones);
        - If a second course of antibiotics is needed: azithromycin PO for 5 days.
    - **If signs of severity**:
        - Parenteral antibiotics (ceftriaxone ± cloxacillin if *S.aureus* is suspected);
        - If a second course of antibiotic is needed: azithromycin PO for 5 days;
        - In addition: **PCP treatment** should be given presumptively to all HIV-exposed or infected children < 1 year of age and any older child with sever immune suppression and not on CTX prophylaxis. For all other HIV-exposed or infected children, it should be considered if there is poor response to broad spectrum antibiotics after 48 h.
f) Clinical response to broad-spectrum antibiotic does not rule out TB. Continue follow up to see if symptoms re-occur.

Supplementary file 2

Standards for Reporting Diagnostic Accuracy Studies (STARD) checklist

Section & Topic	No	Item	Reported #
**TITLE OR ABSTRACT**			
	**1**	Identification as a study of diagnostic accuracy using at least one measure of accuracy<br>(such as sensitivity, specificity, predictive values, or AUC)	Yes
**ABSTRACT**			
	**2**	Structured summary of study design, methods, results, and conclusions<br>(for specific guidance, see STARD for Abstracts)	Yes
**INTRODUCTION**			
	**3**	Scientific and clinical background, including the intended use and clinical role of the index test	Yes
	**4**	Study objectives and hypotheses	Yes
**METHODS**			
Study design	**5**	Whether data collection was planned before the index test and reference standard were performed (prospective study) or after (retrospective study)	Yes
Participants	**6**	Eligibility criteria	Yes
	**7**	On what basis potentially eligible participants were identified<br>(such as symptoms, results from previous tests, inclusion in registry)	Yes
	**8**	Where and when potentially eligible participants were identified (setting, location and dates)	Yes
	**9**	Whether participants formed a consecutive, random or convenience series	Yes
Test methods	**10a**	Index test, in sufficient detail to allow replication	Yes
	**10b**	Reference standard, in sufficient detail to allow replication	Yes
	**11**	Rationale for choosing the reference standard (if alternatives exist)	Yes
	**12a**	Definition of and rationale for test positivity cut-offs or result categories of the index test, distinguishing pre-specified from exploratory	Yes
	**12b**	Definition of and rationale for test positivity cut-offs or result categories of the reference standard, distinguishing pre-specified from exploratory	Yes
	**13a**	Whether clinical information and reference standard results were available to the performers/readers of the index test	Yes
	**13b**	Whether clinical information and index test results were available to the assessors of the reference standard	Yes
Analysis	**14**	Methods for estimating or comparing measures of diagnostic accuracy	Yes
	**15**	How indeterminate index test or reference standard results were handled	Not applicable
	**16**	How missing data on the index test and reference standard were handled	Yes
	**17**	Any analyses of variability in diagnostic accuracy, distinguishing pre-specified from exploratory	yes
	**18**	Intended sample size and how it was determined	yes
**RESULTS**			
Participants	**19**	Flow of participants, using a diagram	yes
	**20**	Baseline demographic and clinical characteristics of participants	yes
	**21a**	Distribution of severity of disease in those with the target condition	Yes
	**21b**	Distribution of alternative diagnoses in those without the target condition	No

	**22**	Time interval and any clinical interventions between index test and reference standard	Not applicable
Test results	**23**	Cross tabulation of the index test results (or their distribution) by the results of the reference standard	Yes
	**24**	Estimates of diagnostic accuracy and their precision (such as 95% confidence intervals)	Yes
	**25**	Any adverse events from performing the index test or the reference standard	No
**DISCUSSION**			
	**26**	Study limitations, including sources of potential bias, statistical uncertainty, and generalisability	Yes
	**27**	Implications for practice, including the intended use and clinical role of the index test	Yes
**OTHER INFORMATION**			
	**28**	Registration number and name of registry	No
	**29**	Where the full study protocol can be accessed	No
	**30**	Sources of funding and other support; role of funders	Yes

Supplementary Figure 1: Participant flow in the study

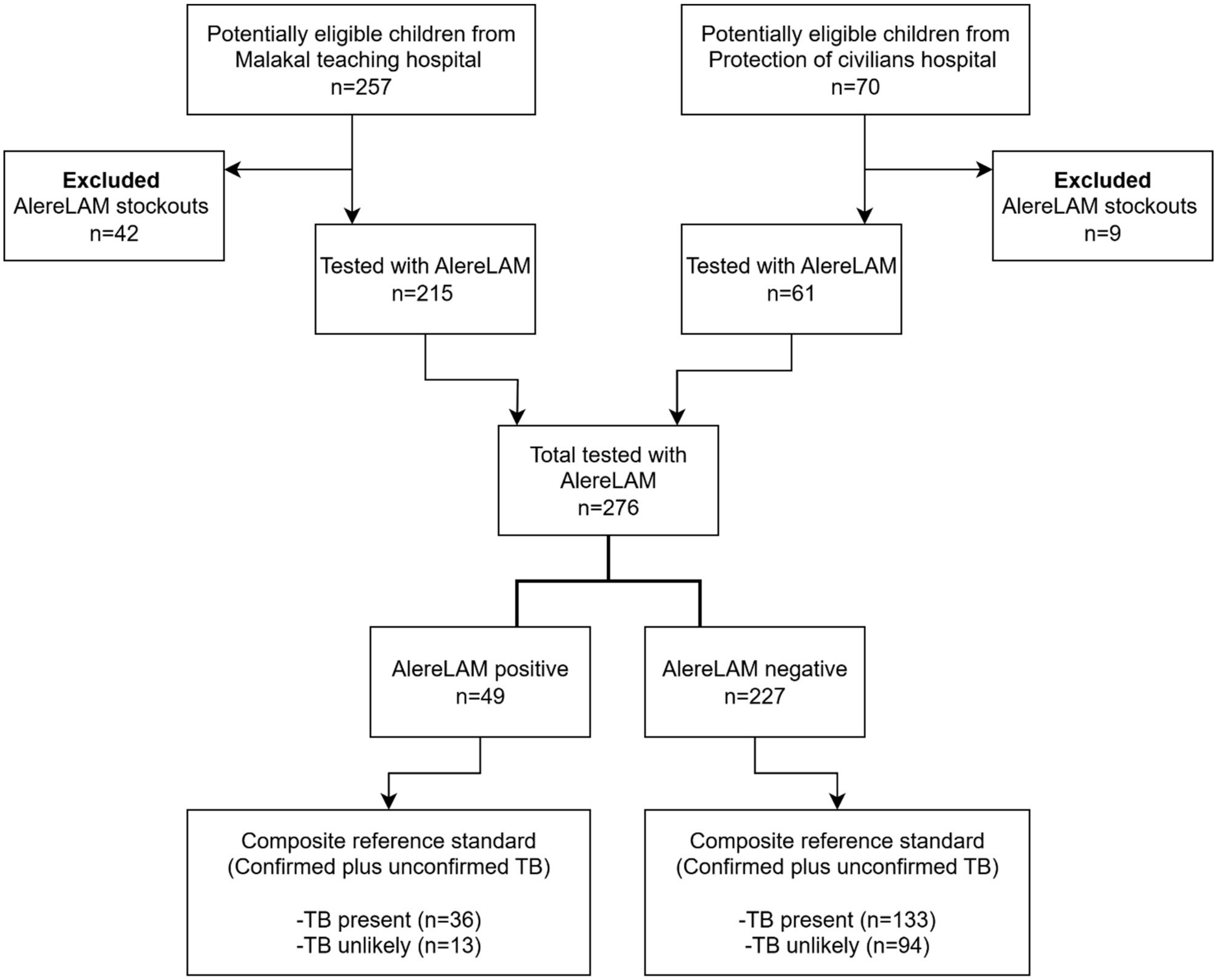

Supplementary table 1: Demographic and clinical characteristics of children by tuberculosis diagnostic category

Characteristic	Overall, N = 276[1]	Confirmed TB, N = 29[1]	Unconfirmed TB, N = 140[1]	TB-total*, N = 169[1]	Unlikely TB, N = 107[1]	p-value[2]
	n (%)	n (%)	n (%)	n (%)	n (%)	
**Age group (years)**						**0.024**
0.5 - <5	160 (58.0)	20 (69.0)	87 (62.1)	107 (63.3)	53 (49.5)	
5 - ≤15	116 (42.0)	9 (31.0)	53 (37.9)	62 (36.7)	54 (50.5)	
median (IQR) age in months	44 (18, 96)	36 (18, 60)	36 (16, 84)	36 (16, 75)	60 (24, 120)	**0.007**
**Gender**						0.2
Male	129 (46.7)	13 (44.8)	61 (43.6)	74 (43.8)	55 (51.4)	
Female	147 (53.3)	16 (55.2)	79 (56.4)	95 (56.2)	52 (48.6)	
**TB history (yes)**	13 (4.7)	1 (3.4)	7 (5.0)	8 (4.7)	5 (4.7)	>0.9
**TB contact**						0.13
No	199 (72.1)	17 (58.6)	101 (72.1)	118 (69.8)	81 (75.7)	
Yes	69 (25)	11 (37.9)	37 (26.4)	48 (28.4)	21 (19.6)	
Unknown	8 (2.9)	1 (3.4)	2 (1.4)	3 (1.8)	5 (4.7)	
**Weight loss (yes)**	256 (92.8)	27 (93.1)	132 (94.3)	159 (94.1)	97 (90.7)	0.3
**Nutritional status**						**0.002**
SAM	179 (64.9)	18 (62.1)	96 (68.6)	114 (67.5)	65 (60.7)	
MAM	63 (22.8)	6 (20.7)	22 (15.7)	28 (16.6)	35 (32.7)	
Normal	34 (12.3)	5 (17.2)	22 (15.7)	27 (16.0)	7 (6.5)	
**median (IQR) MUAC**	135 (115, 150)	130 (118, 146)	130 (112, 148)	130 (113, 148)	140 (120, 161)	**0.002**
Unknown	1	0	1	1	0	
**CLHIV**	26 (9.4)	0 (0)	25 (17.9)	25 (14.8)	1 (0.9)	**<0.001**
**CD4 count (cells/µL)**						0.3
<200	3 (11.5)	0 (0)	3 (12.0)	3 (12.0)	0 (0)	
≥200	17 (65.4)	0 (0)	17 (68.0)	17 (68.0)	0 (0)	
Missing data	6 (23.1)	0 (0)	5 (20.0)	5 (20.0)	1 (100)	
**ART status**						>0.9
On ART	2 (7.7)	0 (0)	2 (8.0)	2 (8.0)	0 (0)	
Not on ART	24 (92.3)	0 (0)	23 (92.0)	23 (92.0)	1 (100)	
**TB type**						>0.9
Pulmonary	75 (44.4)	9 (31.0)	66 (47.1)	75 (44.4)	NA	
Extrapulmonary	41 (24.3)	5 (17.2)	36 (25.7)	41 (24.3)	NA	
Disseminated	53 (31.4)	15 (51.7)	38 (27.1)	53 (31.4)	NA	
**Baseline symptoms**						
Cough	199 (72.1)	21 (72.4)	101 (72.1)	122 (72.2)	77 (72.0)	>0.9

Characteristic	Overall, N = 276[1]	Confirmed TB, N = 29[1]	Unconfirmed TB, N = 140[1]	TB-total*, N = 169[1]	Unlikely TB, N = 107[1]	p-value[2]
Tachypnoea	4 (1.4)	0 (0)	3 (2.1)	3 (1.8)	1 (0.9)	>0.9
Hypoxemia (SpO_2 < 92%)	3 (1.1)	0 (0)	2 (1.4)	2 (1.2)	1 (0.9)	>0.9
Fever (axillary temperature >38°C)	256 (92.8)	28 (96.6)	128 (91.4)	156 (92.3)	100 (93.5)	0.7
Angular deformation of the spine (gibbous)	8 (2.9)	1 (3.4)	7 (5.0)	8 (4.7)	0 (0)	**0.025**
Lymph nodes	29 (10.5)	7 (24.1)	15 (10.7)	22 (13.0)	7 (6.5)	0.087
Subacute meningitis	3 (1.1)	0 (0)	2 (1.4)	2 (1.2)	1 (0.9)	>0.9
Abdomen distended or tender / with ascites	23 (8.3)	1 (3.4)	17 (12.1)	18 (10.7)	5 (4.7)	0.08
> 2 weeks of diarrhoea	39 (14.1)	2 (6.9)	25 (17.9)	27 (16.0)	12 (11.2)	0.3
Painless enlarged Joints	2 (0.7)	0 (0)	2 (1.4)	2 (1.2)	0 (0)	0.5
Pleural effusion	0 (0)	0 (0)	0 (0)	0 (0)	0 (0)	
**AlereLAM positive**	49 (17.8)	8 (27.6)	28 (20.0)	36 (21.3)	13 (12.1)	0.053
**AlereLAM grading**						
Grade 0 (negative)	227 (82.2)	21 (72.4)	112 (80.0)	133 (78.7)	94 (87.9)	
Grade 1	23 (8.3)	3 (10.3)	12 (8.6)	15 (8.9)	8 (7.5)	
Grade 2	16 (5.8)	3 (10.3)	8 (5.7)	11 (6.5)	5 (4.7)	
Grade 3	7 (2.5)	1 (3.4)	6 (4.3)	7 (4.1)	0 (0)	
Grade 4	3 (1.1)	1 (3.4)	2 (1.4)	3 (1.8)	0 (0)	

SAM: Severely acute malnourished; MAM: Moderately acute malnourished; CLHIV: Children living with HIV; ART: Antiretroviral therapy; NA: Not applicable.

[1] n (%); Median (IQR)

[2] P-values were calculated using Pearson's Chi-squared test, Kruskal-Wallis rank sum test, or Fisher's exact test to compare TB-positive and unlikely TB groups.

*TB total= Confirmed TB plus unconfirmed TB

Supplementary table 2: AlereLAM sensitivity in children using positive reference standard of children with confirmed TB (i.e., Xpert-Ultra positive).

	TP / FN	% Sensitivity (95% CI)
**Overall (n=29)**	8 / 21	27.6 (12.7, 47.2)
**Age group**		
<5 years (n=20)	6 / 14	30 (11.9, 54.3)
5-15 years (n=9)	2 / 7	22.2 (2.8, 60)
**HIV status**		
CLHIV (n=0)	0 / 0	NE
Without HIV (n=29)	8 / 21	27.6 (12.7, 47.2)
**Tuberculosis site**		
Pulmonary (n=9)	3 / 6	33.3 (7.5, 70.1)
Extrapulmonary (n=5)	2 / 3	40 (5.3, 85.3)
Disseminated (n=15)	3 / 12	20 (4.3, 48.1)
**Nutritional status**		
SAM (n=18)	5 / 13	27.8 (9.7, 53.5)
Not SAM (n=11)	3 / 8	27.3 (6, 61)

TP: True positives, FN: False negatives; CI: Confidence interval; n: Number of samples; NE: Not estimable; CLHIV: Children living with HIV; SAM: Severely acute malnourished
